# Recent Advances in Liquid Biopsy in Patients With Castration Resistant Prostate Cancer

**DOI:** 10.3389/fonc.2018.00397

**Published:** 2018-09-24

**Authors:** Vincenzo Di Nunno, Lidia Gatto, Matteo Santoni, Alessia Cimadamore, Antonio Lopez-Beltran, Liang Cheng, Marina Scarpelli, Rodolfo Montironi, Francesco Massari

**Affiliations:** ^1^Division of Oncology, S.Orsola-Malpighi Hospital, Bologna, Italy; ^2^Oncology Unit, Macerata Hospital, Macerata, Italy; ^3^Section of Pathological Anatomy, School of Medicine, United Hospital, Polytechnic University of the Marche Region, Ancona, Italy; ^4^Department of Pathology and Surgery, Faculty of Medicine, Cordoba, Spain; ^5^Department of Pathology and Laboratory Medicine, Indiana University School of Medicine, Indianapolis, IN, United States

**Keywords:** prostate cancer, metastatic castration resistant prostate cancer, CTCs, liquid biopsy, circulating DNA

## Abstract

Management of localized and advanced prostate cancer benefits from several therapeutic options with a surprising improvement in terms of clinical outcome. The selection of patients more likely to benefit from a specific approach still remains a key issue as well as the early identification of patients with aggressive disease which could benefit from a more aggressive treatment strategy. The lack of reliable bio-marker in castration resistant setting able to monitor response to treatment and early inform about tumor progression is an emerging issue. Accordingly, circulating DNA and circulating tumor cells appears a promising and attractive approach despite to date practical applications of these techniques are few and not validated. The aim of this review of the literature is to explore current knowledge on liquid biopsy in prostate cancer focusing on possible future applications.

## Introduction

Prostate Cancer (PCa) represents the most common adult malignancies ranking as one of the major cause of cancer related death in men (1). Management of the disease accounts various options in both localized and advanced stages. Each options are generally evaluated according to different variables related to patients (performance status, comorbidities, disease related symptoms, and patients' preferences) and tumor features (biological aggressiveness and site and number of metastases). Thus, the management of localized stages could range from a first instance no invasive approach (watchful waiting or active surveillance approach) to a radical approach by surgery, external radiation treatment, a combination of both of them (radiation treatment in case of positive surgical margins) or also brachytherapy (which consists on the prostate implantation of sealed radiotherapy sources) with or without an adjuvant androgen deprivation therapy (ADT) (2–8).

Similarly, advanced stages of the disease count different therapeutic options. As first approach ADT represents the cornerstone of advanced prostate cancer due to the high sensitivity of tumor cells to hormone deprivation. The addiction of further treatment including anti-androgens abiraterone acetate or docetaxel can improve the outcome of patients with metastatic castration sensitive prostate cancer (mCSPC) (9–15).

After a first period of hormone deprivation sensitivity, tumor cells develop several mechanisms which lead to overcome the hormone inhibition leading to metastatic castration resistant prostate cancer (mCRPC). In this setting, several different agents have demonstrated to be effective treatment: new hormonal agents (abiraterone, enzalutamide, apalutamide), chemotherapy (docetaxel, cabazitaxel), radiometabolic drugs (Radium 223), and Sipuleucel-T immunotherapy (16–25).

## Rational for liquid biopsy in prostate cancer

The availability of several active therapeutic options has led to different emerging needs in clinical practice requiring the development of reliable markers able to monitor response to treatment and help clinicians to select patients more likely to benefit from one approach rather than another.

Prostate-Specific Antigen (PSA) represents a reliable and useful biomarker adopted for early detection and early diagnosis of disease recurrence progression. However, it does not give information about biological features of the disease and it loses its predictive rule in mCRPC setting (26).

Liquid biopsy is an emerging technique which purposes is the detection of tumor cells/tumor DNA from patients' peripheral blood.

There are several issues which make the development of liquid biopsies in prostate cancer an attractive approach: (1) the low invasiveness; (2) the early detection of more aggressive tumors since early phases;(3) the early diagnosis of residual tumors or micro-metastases after surgery. (4) the monitoring of tumor response/progression to systemic treatment in advanced setting of the disease and especially in mCRPC; (5) the prediction of tumor sensitivity/resistance to systemic treatments; (6) the acquisition of an accurate genetic assessment of the disease focusing on key alterations which are related to tumor resistance. In particular, several genomic alterations seem to be attractive target due to their correlation to treatment resistance and/or sensitivity to specific treatments (27–30). Some of the more attractive targets are:

- **Phosphate and tensin homolog (PTEN) loss**. PTEN loss results in PI3K/AKT activation which has been associated to worst survival due to higher tumor proliferation and resistance to hormonal treatment. The inhibition of the PI3K/AKT/mTOR pathway could be an interesting target in this subgroup of patients which could be associated to an Androgen Receptor (AR) inhibition (31, 32).
- **MYC** amplification is generally acquired in metastatic phases of the disease and is correlated to poor prognosis and higher Gleason score. Furthermore, more than one evidences seem to correlate the combination of MYC amplification and PTEN loos to worst prognosis and increase risk of tumor related death (33, 34).
- **Androgen Receptor (AR) mutations** and in particular AR splice variant 7 (AR-V7) is known to be related to resistance to hormonal treatments including also new hormonal agents abiraterone and enzalutamide (35).
- **TMPRSS2-ERG** gene fusion leads to ETS-related gene (ERG) and steroidogenic enzyme AKR1C3 co-overexpression which promotes AR signaling and represents a promising target in prostate cancer (36, 37).
- **DNA repair genes deficiency** and in particular genes related to the identification of single strand breaks (such as PARP1 and PARP2) as well as the identification of the alterations of non-homologous recombination system genes (such as BRCA1, BRCA2, PALBB2, MRE11, Check2, RAD51, XRCC2/3) appears an attractive approach for two reason. First, tumors with repair genes deficiency are related to more aggressive features and poorer survival. Second, therapeutic implications related to these genomic assessments involve a possible sensitivity to platinum cytotoxic therapy. The development of PARP inhibitors represents another possible target for the management of advanced prostate cancer which has already been evaluated in small trials and is currently under clinical investigation (38–43).

Due to these issues, the development of reliable techniques able to perform liquid biopsy appears a promising and suggestive approach (Table 1). Here we performed a review of the main techniques adopted or under investigation focusing our attention on approaches based on circulating tumor cell (CTC) and circulating DNA (ct-DNA) detection. Table 2 summarizes the current methods available for CTC detection as well as the percentage of detection (See also below).

**Table 1:** An overview of ongoing clinical studies evaluating CTCs/ctDNA in prostate cancer patients.

**Trial**	**Patients enrolled**	**Study description/outcomes**
NCT03284684	Patients undergoing surgery for non-metastatic solid tumors: colon, breast and prostate.	Change in concentration of total mutant circulating DNA. Change in proportion of mutant circulating DNA. Change in integrity index of circulating DNA for ACTB gene. Change in integrity index of circulating DNA for KRAS gene.
NCT02449837	Patients undergoing radiation treatments for one of six cancer types including PC.	To measure CTCs levels to evaluate the change pre- and post-treatment. Change in CTC levels from Baseline to Post-RT treatment and the correlation with local tumor response or pathological evaluation
NCT01961713	Subjects with prostate cancer diagnosed on prostate biopsy who undergo radical prostatectomy	To evaluate the relationship between pre-operative CTC quantity and pathologic stage in men with early stage prostate cancer undergoing prostatectomy. To examine the relationship between persistent CTCs and biochemical recurrence after radical prostatectomy for localized prostate cancer
NCT02997709	Men with intermediate to high risk prostate cancer who are candidates for radiotherapy (RT)	Comparison of Pre- and Post-Treatment Quantitative Imaging Parameters to Changes in Circulating Tumor Cells Over Time in Prostate Cancer Patients Receiving Radiation Therapy (RT) with or without Androgen Deprivation Therapy per standard of care
NCT02853097	Prostate cancer patients at various points throughout androgen deprivation therapy and at the initiation of androgen deprivation therapy, enzalutamide, abiraterone and docetaxel.	To document the appearance of androgen receptor isoform splice variant 7 (AR-V7) expression over the course of therapy in castration-resistant prostate cancer (CRPC). To determine whether detectable AR-V7 is associated with a shortened duration of treatment benefit of abiraterone or enzalutamide.
NCT03089099	mCRPC	To determine whether sequentially analyzing the expression of molecular markers in high volume circulating tumor cells in metastatic castration-resistant prostate cancer patients can predict the therapeutic effects and outcomes of these patients.
NCT03488706	Prostate cancer screening with PSA is plagued by high rate of unnecessary prostate biopsies, especially in the “gray zone” (PSA levels: 4.00 ng/ml e 10.99 ng/ml)	Circulating tumor cells detection Using a circulating-tumor-cell (CTC) test to detect prostate cancer in patients in the PSA “gray zone” level
NCT03236688	mCRPC	Demonstrate detection of ARv7 splice variant transcripts from exosomes in the circulation of MCRPC patients pre and post treatment with selective Androgen pathway inhibitors (i.e., abiraterone and enzalutamide)
NCT02771769	Patients with planned prostate biopsy	Multi-center prospective study in which blood samples will be taken from 1500 male patients aged between 21–80 scheduled for prostate biopsy. Analysis of cell-free cancer DNA extracted from these samples will be undertaken to determine whether copy number instability scores derived from the cfDNA correlates with PSA screening levels and prostate biopsy results (i.e., Gleason score) in these patients
NCT02723526	Patients with newly Diagnosed Metastatic Hormone-Sensitive Prostate Cancer	To determine whether sequentially analyzing the expression of tumor markers in circulating tumor cells in newly diagnosed metastatic hormone-sensitive prostate cancer patients can predict the outcome of these patients.
NCT02742259	Metastatic prostate cancer to the bone	Confirmation of the clinical utility of the cutoff level for the Prostate Cancer Assay for prognosis of progression free survival (PFS) in comparison to the predicate device, CellSearch CTC Assay
NCT02456571	Metastatic PC	To explore the prevalence of expression of four immune checkpoint biomarkers on circulating tumor cells (CTCs) from men with metastatic prostate cancer
NCT02735252	Metastatic PCa.	Develop a first-in-man CTC-based molecular taxonomy of CRPC. Comparison of median PFS to CTC-based AR-v7 status.
NCT02099864	Advanced PCa patients receiv- ing enzalutamide therapy.	Correlation between PSA response and gene expression signatures, DNA copy number alterations, mutations. Assess the association for changes in CTC counts from baseline and maximal PSA observed while on study.

**Table 2:** An overview of CTCs detection techniques.

**Method**	**Mechanism**	**CTCs detection rate/ other outcomes**	**Limitations**
CELLSEARCH System (44)	A 7.5 mL sample of blood is placed in a special tube, centrifuged to separate solid blood components from plasma, then placed in the CELLTRACKS® AUTOPREP® System. Cells binds ferro-fluid nanoparticles presenting antibodies targeting epithelial adhesion molecules, then CTCs are magnetically separated from other blood cells. CTCs are stained with cytokeratin monoclonal antibodies, DAPI (a DNA stain) and leukocytes which may have contaminated the sample are marked by antibody targeting CD45. Stained CTCs are then placed onto Cell-Spotter Analyzer (a four-color semi-automated fluorescence microscope) for the CTCs enumeration CTCs+: DAPI+, cytokeratine +, CD45—cells	CTCs detected in 57% of patients with prostate cancer	- Low CTCs detection rate in non-metastatic prostate cancer - Conflicting results about correlation between CTCs number and treatment response.
CELLCOLLECTOR EPISPOT (45)	Cell-Collector is based on a sterile stainless steel medical wire, covered with 2 μm gold and a hydrogel layer which is covalently coupled with antibodies against the EpCAM protein and pan-keratins. CD45 staining (performed to exclude unspecific leucocytes) CTCs +: CTCs identified as pan-keratin positive, leukocyte marker CD45 negative. EPISPOT on an EpCAM-independent enrichment method (i.e., leukocyte depletion) and enables the identification of viable PSA-secreting tumor cells CTCs+: PSA+ cells.	Combining Cellsearch, CellCollector and Epispot assay, detection rate of CTCs was 81.3%	- Experimental approach. - This approach does not offer a characterization of CTCs. - Impact on prognosis and predictive value under investigation.
Microfluidic capture of CTCs (46).	Considering the expression of PSA (up-regulated by AR) and PSMA (down regulated by AR) they classified CTCs in AR on, AR mixed and AR positive according to the expression of PSA (+ in AR on and mixed) and PSMA (+ in AR off and mixed).	CTCs detection rate: 80%	- Experimental approach - Under investigation for detection of anti-androgen resistance mechanisms.
EPCAM cells enrichment and sequencing (47).	The recovered cells, enriched with CTCs, were deposited into dense arrays of subnanoliter wells and imaged by automated epifluorescence imaging. Enrichment was obtained through Illumina MagSweeper CTCs expressing EpCAM. Individual EpCAM (+) CD45 (–) CTCs were recovered by robotic micromanipulation for whole genome amplification using multiple displacement amplification.	Mutation concordance between CTCs and primary or metastatic tumor tissue: 86%	- Experimental approach. - High cost. - Loss of concordance between CTCs mutation and primary/metastatic tumor tissues.
ADNAtest (48).	Is a device able to isolate MUC1-negative and EpCAM positive CTCs. After CTCs isolation, cells are lysed and RNA is extracted for downstream analyses with RT-PCR. Of note this device adopts primers against EGFR, PSA and PSMA making a sample positive if one of these genes are expressed.	CTCs detection rate: 62%	- Experimental approach - High cost - Few data about the application of this approach in localized/ non metastatic prostate cancer.

## Circulating tumor cells in prostate cancer

To date, the CellSearch system assay is the only FDA approved method for the detection of CTCs in prostate cancer (Table 2). This device consists of different components including a CellPrep system which is a semi-automated sample preparation system and a CellSearch Epithelial Cell Kit. This last component involves ferro-fluids coated with epithelial cell-specific EpCAM antibodies and a mixture of antibodies directed against cytokeratins 8, 18, 19, CD45 conjugated to allophycocyanin and DAPI (nuclear dye 4', 6 diamidino-2-phenylindole for fluorescent cells label). After an incubation period in which CTC are isolated from peripheral blood and enriched in EpCAM composed ferro-fluids the MagNest Cell Presentation Device (a device composed of a chamber with two magnets) orients labeled cells for analysis in a CellSpotter Analyzer (a four-color semi-automated fluorescence microscope) for the CTCs enumeration (44).

Initial studies carried out on patients with different solid tumor demonstrated a promising activity with this method and regarding patients with PCa detection of CTCs was possible in 57% of patients (44).

Further study aimed to investigate the clinical value of CTCs detected by CellSearch assay showed that CTCs baseline levels were an independent prognostic factor for overall survival (OS) (49).

In 2008, de Bono et al. identified a correlation between CTCs number and median overall survival. In this study carried out on 231mCRPC two distinct subgroup of patients were identified: one (Unfavorable group) which showed a CTCs number of 5 or more and the other (favorable) with < 5 CTCs per 7.5 mL of blood. Overall survival was significantly better in favorable group (21.7 vs. 11.5 months). Moreover, patients who presented a significant decrease of CTCs number during or after treatment (moving from unfavorable to favorable groups) significantly improved their survival compared to patients who continued to present a CTCs number of 5 or more CTCs. According to the results of de Bono et al, a meta-analysis of 10 studies confirmed the prognostic rule of CTCs in patients with prostate cancer (50).

Furthermore, pre-planned analyses of large phase III trials: SWOG 20421 (docetaxel with or without atrasentain in mCRPC patients), COU-AA-301 (in which a score composed by LDH levels and CTCs divided patients in 3 different subgroups with favorable, intermediate and poor prognosis) and AFFIRM (enzalutamide in patients with mCRPC progressed to chemotherapy) confirmed the prognostic rule of CTCs as independent factor related to OS (51–53).

Unfortunately, none of these studies demonstrated an association between CTCs number and response to treatment and so the role of CTCs in this setting still remains unclear. Moreover, another possible issue which could partially explain the failure of this approach in clinical practice is the low detection rate of CTCs in non metastatic patients which ranges only from 5 to 27% (54). To avoid this problem, Kuske et al combined three different methods for the detection of CTCs before and after prostatectomy in non metastatic patients with PC, CellSearch system assay, CellCollector (a system capturing EpCAM-positive CTCs by an antibody-coated needle introduced in arm vein) and EPISPOT (a system able to enrich CTCs by negative depletion of leukocytes and detects circulating prostate cancer cells thanks to their active secretion of PSA) (45). CTCs were detected in 37, 54.9 and 58.7% of patients using CellSearch, CellCollector, and EPISPOT, respectively. The cumulative positivity rate of the three CTC assay was 81.3% and despite it is not a validated approach, it represents an attractive early method able to estimate the risk of tumor recurrence or persistence after surgery.

A combined analysis of COU-AA-301 and IMMC-38 trials showed that an increase of 30% in CTCs count from baseline was independently associated to worst OS in patients treated with abiraterone and chemotherapy (53). To sustain the correlation between CTCs count changes and survival, another analysis performed on 119 patients with CRPC treated at the Royal Marsden Hospital suggested that a decrease of 30% in CTC counts from baseline was associated to improved survival (55).

The only CTCs enumeration resulted in an independent prognostic factor with an unclear role in terms of early diagnosis of disease recurrence/persistence after surgery as well as a predictive response factor. Another interesting approach consists in a characterization of CTCs resulting in a genetic assessment and in a detection of target altered pathways.

AR protein has been extensively investigated in prostate cancer CTCs. Through a FISH based assay AR gene amplification detection in CTCs was possible in 40% of cases, a percentage comparable to the AR amplification described in bone metastases biopsy analyses (47). Further investigations demonstrated that patients with higher cytoplasm expression of AR resulting in a reduction of nuclear translocation was significantly associated to better response to docetaxel (56). By a microfluidic capture of CTCs Miyamoto et al. evaluated dynamic changes in CTCs AR expression. In particular, considering the expression of PSA (up-regulated by AR) and PSMA (down regulated by AR) they classified CTCs in AR on, AR mixed and AR positive according to the expression of PSA (+ in AR on and mixed) and PSMA (+ in AR off and mixed). Moreover, Authors identified that AR status changed from “on” to “off” during ADT while patients treated with abiraterone acetate with an increase of AR-on CTCs or baseline level of AR-mixed more than 10% were significantly associated to worse overall survival (46). The technology developed by Myamoto et al was also adopted for the detection of anti-androgen resistance mechanisms in CTCs demonstrating higher activation of Wnt signaling and considerable heterogeneity in signaling pathways, expression of AR gene mutations and splicing variants (57).

Due to the important role of AR-V7 in mCRPC (35), several studies have focused on the detection of this splice variants on CTCs. An EpCAM assay demonstrated that CTCs-ARV7+ detection was associated to resistance to enzalutamide and abiraterone but no to docetaxel and cabazitaxel and that the detection of these CTCs was independently associated to worse clinical outcome compared to patients with CTCs-ARV7- cells (58–60). Other studies modified the CTCs and ARV7 detection method in order to evaluate the AR-V7 cellular localization (61) and the presence of other splice variants of AR (62). Particularly, not only AR-V7 but also other slice variants of the AR protein were significantly associated to worse progression free survival. Moreover 6 of 17 poor responders to treatment were AR-V7 negative, but carried other AR perturbations (62).

About other pathways detected in CTCs, the PTEN loss assessed by FISH and Epic Sciences test (an assay which adopted a fibreoptic array scanning techniques for the detection of DAPI, CD45, cytokeratins stained cells) has been associated to worse clinical outcomes (63, 64) while the detection of TMPRSS2-ERG fusion gene performed by microfluidic device and by the use of a RT-PCR analysis failed to show a predictive response value to abiraterone acetate in mCRPC patients (47).

Next Generation Sequencing (NGS) involves a series of different techniques able to perform a whole genome sequencing of tumor cells. The possibility to obtain a complete genomic assessment from CTCs appears a novel and promising approach investigated in different studies.

In 2014, Lohr et al evaluated a method able to perform a CTCs isolation, enrichment (throuEp-CAM expressing CTCs), genomic amplification and sequencing in metastatic PC (65). They demonstrated that a complete mapping of the standard exome was possible in CTCs. NGS analysis of CTCs and tumor sample of a single patient with advanced prostate cancer showed a concordance of 86% from the mutations isolated in CTCs and genomic anomalies identified in primary or metastatic tumors (66). Despite NGS performed to CTCs represents an attractive approach, to date no validated or prospective studies have been carried out and so this method is still under investigation.

Another interesting issue is the detection of whole blood RNA, without enriching for CTC. In 2012, Ross et al assessed a whole blood RNA transcript based model as prognostic factor in patients with PC. After the analysis of blood collected from 62 men with mCRPC, they identified a six gene model (genes considered were: ABL2, SEMA4D, ITGAL, C1QA, TIMP1, CDKN1A which are genes involved mainly in immunity regulation) able to divide patients in two risk groups with different mOS (67). In the same year, Olmos et al carried out a validation study of a nine-gene signature as prognostic factor (68). Design of the study consisted in a derivation set in which patients with mCRPC and patients in Active Surveillance were used as case and control groups respectively. After genomic assessment 94 patients were divided in four distinct prognostic groups. Thus nine altered genes HMBS, TMCC2, SLC4A1, STOM, GABARAPL2, RIOK3, TERF2IP, TFDP1) isolated in prognostic groups with worst survival (composed of only mCRPC patients) were validated in a validation set of patients with mCRPC. More recently, an assessment of 5 key genes (KLK3, KLK2, HOXB13, GRHL2, FOXA1) obtained after reverse transcription polymerase chain reaction (RT-PCR) demonstrated to be a reliable prognostic marker compared to CellSearch system count (48). Isolation of two or more of the selected genes were possible in 53% (51 /97) patients with mCRPC. AdnaTest is a technique adopted for CTCs enrichment and consists of a device able to isolate MUC1-negative and EpCAM positive CTCs. After CTCs isolation, cells are lysed and RNA is extracted for downstream analyses with RT-PCR. Of note this device adopts primers against EGFR, PSA, and PSMA making a sample positive if one of these genes are expressed. Sensitivity of KLK2, KLK3, HOXB13, GRHL2, and FOXA1 genes detection by this method is similar to DDPCR (direct detection PCR) and both of these techniques showed a higher sensitivity compared to CellSearch system (69).

Concerning the several devices utilized for CTCs detection, enrichment and evaluation, only CellSearch has been approved from FDA. However, despite a large range of potential applications (such as diagnosis, evaluation of treatment response, early detection of tumor relapse, and progression) CTCs detection by CellSearch is not commonly adopted in clinical practice. This mainly due to a low sensitivity of the method as well as a conflicting relationship between CTCs and treatment response evaluation. Several other approaches are under investigation. It is likely that CTCs evaluation will be an important factor able to improve our decisions in clinical practice (48, 69).

## Circulating DNA in prostate cancer

The evidence that cell-free DNA could be detected in peripheral blood is a well known issue, and its application in clinical practice has been investigated only in last years. Regarding cancer patients, the unique composition of tumors' ctDNA presenting several genomic mutations (especially single base-pair substitution) which are not detectable in ctDNA originating from normal cells make tumor ctDNA an ideal markers of the disease. Moreover, the possible correlation between mutations detected on ctDNA and genomic mutations of primary or metastatic tumors make cDNA a unique markers able to provide key information by a no-invasive approach.

Regarding PC, ctDNA could be detected in peripheral blood and detection of known driver aberrations can be obtained in more than 97% of cases. Moreover, changings in ctDNA genomic mutations could be detected by repeated analyses of ctDNA with high grade of concordance with genomic assessment of primary tumors or metastases (70, 71).

The quantitative assessment of ctDNA has been related to prognosis of patients with PC in different studies (72, 73). In particular, Romanel A et al exanimated AR status of mCRPC patients starting abiraterone acetate. They detected a 45% of patients (tot number 97) with AR point mutations (T878A/L702H) before the first administration of abiraterone who showed a significant worse overall survival (73). Similarly, other studies confirmed the prognostic role of AR genomic alterations as prognostic markers raising the acquisition of ctDNA examination as a possible to monitor response to hormonal agents and to achieve an early diagnosis of progressive disease (74–77).

As known, mutation in DNA repair genes is acquiring an increasing interest in PC due to the association by these mutation and more aggressive tumor features and to the possible benefit derived from a PARP targeted treatment. Mutations in repair genes are common in prostate cancer. In a DNA assessment of 692 men with metastatic PC a total of 84 germiline DNA repair gene mutations (BRCA2, ATM, CHEK2, BRCA1, RAD51D, and PALB2) were found in 82 men (78). This study demonstrated that incidence of germline mutations of DNA-repair gene were common (as detected in 11.8% of all patients) in metastatic patients regardless to age and family history of prostate cancer.

PARP inhibition is one of the important strategy currently under investigation in patients with metastatic prostate cancer. In a phase II trial 50 mCRPC patients received the PARP inhibitor Olaparib (41). 17 (33%) patients showed an objective response while NGS sequencing showed that 16 patients presented homozygous delections, deleterious mutations, or both in DNA repair genes (BRCA1/2, ATM, Fanconi's anemia genes and CHECK2). A subsequent analysis of tumors DNA highlighted that patients with an overall reduction of 50% or more of ctDNA were associatied with better OS and PFS (79). In ASCO 2018, Clarke et al presented the results of a phase II study comparing the administration of Abiraterone with Olaparib or placebo in 171 patients with mCRPC (80). This trial met its primary endopoint showing a better radiological PFS in patients receiving olaparib. Of note Authors researched homologous recombination repair mutations by a NGS approach in tumor samples and plasma. Sequencing was possible on 91 of 136 patients and positive results (defined as discovery mutated patients) was obtained in 13 patients. By germline analysis and tumor sample analyses detection of homologous recombination repair mutations were identified in 3 patients (38 tumor samples analyzed of 68 total samples) and 7 patients (by a germline analysis of 102 patients). Results of this study raising the dosage and analyses of cDNA as possible approach for the detection of keys DNA-repair gene mutations. Other larger prospective trials are needed to explore the role of ctDNA in this setting.

Another promising target gene is represented by PTEN loss which has show to be a predictive biomarker of response to treatments targeting PI3K/AKT pathway. Hyper-activation of the PI3K/Akt/mTOR resulting from PTEN loss is related to decreased AR transcription output and stability and vice versa. The addiction of ipatasertib (an Akt inhibitor) to abiraterone acetate increased radiological PFS of patients with mCRPC and PTEN loss previously treated with docetaxel based therapy and progressed during at last one previous hormonal therapy (81).

Extracellular vesicles are membrane-enclosed structures that are released from all cells in the body. These vesicles contain several substances such as proteins, lipids, RNA, and DNA and are considered a very promising tumor-related biomarkers. Recently, it has been demonstrated that large extracellular vesicles isolated from plasma of patients with prostate cancer cells are an important source of chromosomal DNA which reflects faithfully genetic aberration of the cell of origin, including copy number variations of genes frequently altered in metastatic prostate cancer (such as MYC and PTEN) (82). The study of extracellular vesicles represents a novel and promising approach for biomarkers development in prostate cancer however further studies are needed to explore the effective value of this method.

## Conclusion

The surprising potential of CTCs or tumors' ctDNA detection, characterization and genomic assessment have start a revolution which probably will give important results in next years. Despite to date application of these techniques are few probably that better knowledge of genomic anomalies of PC and their correlation with the clinical course of the disease as well as their potential relationship with specific targeted treatments will increase the attention on this issue.

## Author contributions

RM and FM: conception and design; VD and LG: drafting the manuscript; MSa and AC: review of the literature; LC, MSc, and AL-B: critical revision of the manuscript.

### Conflict of interest statement

The authors declare that the research was conducted in the absence of any commercial or financial relationships that could be construed as a potential conflict of interest.
